# Bayesian partially-protected regularization as a model selection tool

**DOI:** 10.1080/02664763.2025.2559025

**Published:** 2025-10-07

**Authors:** Yasir Atalan, Selim Yaman, Jeff Gill

**Affiliations:** a Department of Government, American University, Washington, DC, USA; b Center for Social Data Science, University of Mannheim, Mannheim, Germany; c Departments of Government, Mathematics/Statistics, Center for Data Science, American University, Washington, DC, United States

**Keywords:** Lasso, elastic net, regularization, Bayesian statistics, predictive modeling, machine learning

## Abstract

This work first describes Bayesian Partially-Protected Lasso (BPL), which combines the power of Bayesian Lasso with the ability to protect key theoretical explanatory variables from shrinkage to a zero effect in the model. This approach allows researchers to identify protected and non-protected variables so that data with many explanatory variables can be efficiently machine-explored without sacrificing theoretically important predictors. We provide the statistical background, algorithms, examples, and easy to use tools in an R package. We then introduce BPEN – Bayesian Protected Elastic Net estimation process that builds on the idea of the Bayesian Partially-Protected Lasso. Since the Elastic Net adds a second penalty term to the standard Lasso it provides a more flexible regularization process. This is a novel approach that combines the robustness of the Elastic Net in sifting through potentially large sets of variables while simultaneously safeguarding the integrity of those grounded in theoretical principles.

## 1. Introduction

There are now a staggering number of powerful tools that have been or can be imported from the broad spectrum of data science where the objective is typically categorical prediction rather than fealty to the history of a specific literature. This is at odds with conventional empirical work in the social sciences, which historically focuses on using theoretically driven explanatory factors with a strong history that are then used to build generalizable models adding to a specific line of inquiry. Yet, a profusion of machine learning based tools are now routinely adopted as the primary methodology across nearly every subfield of the discipline, often without thoughtful consideration of the important epistemological consequences. This leads to a tension in modern empirical social science that has yet to be fully resolved in a meaningful and constructive way.

The tools associated with machine learning and artificial intelligence are permeating every academic field, and the rate is increasing dramatically right now. Many of these methods are classifiers that take, possibly very large, datasets and use within-data characteristics to classify cases into predefined or computer-defined categories, as opposed to more conventional analytics that require humans to establish criteria. An example of the latter is a standard logit regression model wherein a researcher selects a set of explanatory variables and then classifies on the predicted outcomes separating at 0.5 as in the classic naive-criteria two-by-two summary table. Are social science researchers willing to abandon this tried-and-true strategy for binary classification with strictly machine-based decisions if those decisions result in notably better classification? The answer is almost certainly *no* given richness of the literatures we draw from in these fields, but are researchers also willing to ignore the startling power of current and emerging machine learning tools? The answer is almost certainly *no* to this question as well.

In this work we cautiously introduce a model selection tool from machine learning adding a new and useful feature that allows protection for theoretically important explanatory variables while at the same time harnessing the stunning power of recent data science tools to deal with very big data. The Bayesian Partially-Protected Lasso (BPL) is a recent approach that combines the robustness of the statistical Lasso in sifting through potentially enormous amounts of explanatory variables while simultaneously safeguarding the integrity of those grounded in theoretical principles. We explore the mathematical foundations of the BPL method for both interval and dichotomous outcomes, extending the Bayesian Lasso model by integrating different prior distributions for protected and non-protected variables, thereby preserving selected variables during the Lasso shrinkage process. We then extend the novelty of that approach to the more flexible Elastic Net setup to provide a powerful version that accomplishes the same goals but with more power. The utility of this semi-protected approach is demonstrated using simulation as well as re-analyses of published social science works employing the Lasso and the Elastic Net. This approach is also intended to be a model for introspective introduction of assertive methodologies from data science in to applied empirical social science research.

## 2. Bayesian partially-protected regularization

As in the standard linear model setup, let X be the n×p matrix of standardized predictors with a leading column of ones, y the *n*-length outcome variable vector, β the *p*-length vector of regression coefficients to be estimated, and $\sigma^{2}$ the residual variance, but add $\lambda^{2}$ as penalty term for overfitting with too many covariates. The Least Absolute Shrinkage and Selection Operator [25] is given by:

(1) $$\begin{array}{r} {\hat{\beta }}_{\text{L}}=\arg\min_{\beta }\left[{\left(y-\mathrm{X\beta}\right)}^{\prime }\left(y-\mathrm{X\beta}\right)+\lambda \sum_{j=1}^{p}|\beta_{j}|\right], \end{array}$$

which produces the best set of non-zero coefficient estimates when the goal is exclusively predicting the y outcomes with the resulting predictions $\hat{y}={\hat{\beta }}_{\text{L}}X$. The remaining potential predictors are shrunk by the Lasso to zero and are therefore discarded in this prediction calculation. While the Lasso regression estimator has optimal prediction properties under general conditions,it disregards regressors that are theoretically important in a given literature, hence our motivation for a protection mechanism. Unfortunately with the non-Bayesian Lasso valid (consistent) standard errors for the terms shrunk to zero are not produced by this process even with the use of bootstrapping, and several awkward workarounds have been suggested.

The advantages of the Bayesian version of the Lasso were forcefully argued in [22] and [15] starting with specifying a general version of the Laplace (double-exponential) distribution for $\sigma^{2}$ to get a prior distribution of the form:

(2) $$\begin{array}{r} \pi (\beta \;|\;\sigma^{2})=\prod_{j=1}^{p}\frac{\lambda }{2\sqrt{\sigma^{2}}}\;{\text{e}}^{-\lambda |\beta_{j}|/\sqrt{\sigma^{2}}} \end{array}$$

These advantages include estimating the penalty term within the MCMC sampler, generation of valid standard errors for the parameters shrunk to zero, and easy extensions to more elaborate forms like the Elastic Net and hierarchical specifications. Conditioning on $\sigma^{2}$ guarantees that the posterior form is unimodal, which improves the mixing of the MCMC sampler and provides more interpretable results. Bayesian Lasso has been a popular choice from social sciences to gene selection [1,2,4].

The protected version introduces the *p*-length $\tau^{2}$ hyperparameter vector as a Bayesian precision term and specifies the priors:

(3) $$\begin{array}{r} \epsilon_{i}∼N(0,\sigma^{2}),\;i=1,\ldots ,n\;\beta_{j}∼N(0,\tau_{j}^{2}\sigma^{2}),\;j=1,\ldots ,p\;\lambda^{2}∼\Gamma (1,0.1). \end{array}$$

We can then separate $\tau^{2}$ into protected ( $\tau_{\text{protected}}^{2}$) and non-protected ( $\tau_{\text{non-protected}}^{2}$) groups, leading to the additional priors:

(4) $$\begin{array}{r} \tau_{\text{non-protected}}^{2}∼\text{exp}(\lambda^{2}/2)\;\tau_{\text{protected}}^{2}∼\Gamma (1,1) \end{array}$$

Note that the $\tau^{2}$ prior specification for the protected variables above does not contain the penalty term, thus saving it from Lasso shrinkage. To derive the marginal posterior distributions from this setup, we calculate the full conditional distributions, facilitating iterative draws of new values for each parameter given the current values of the other parameters with a Gibbs Sampler. Performing these steps iteratively many times produces empirical marginal posterior draws after convergence of the Markov chain can be asserted. All code is written in R and standard diagnostics, both graphical and formal, are used to assess the state of the chain with regard to its stationary distribution. The Bayesian Lasso for interval measured outcomes with partially protected variables uses the standard semi-conjugate specification, and is estimated by the following sampling scheme:

**Update** β: $\beta |\tau^{2},\sigma^{2},Y∼N((\frac{1}{\sigma^{2}}X^{T}Y+\text{diag}(\frac{1}{\tau^{2}}){)}^{-1}X^{T}Y,\sigma^{2}(\frac{1}{\sigma^{2}}X^{T}X+\text{diag}(\frac{1}{\tau^{2}}){)}^{-1})$
**Update** $\tau^{2}$ For non-protected variables: $\tau_{\text{non-protected}}^{2}|\beta ,\sigma^{2},\lambda^{2}∼\mathrm{IG}(\sqrt{\frac{\lambda^{2}\sigma^{2}}{\beta_{\text{non-protected}}^{2}}},\lambda^{2})$
For protected variables: $\tau_{\text{protected}}^{2}|\beta ,\sigma^{2}∼\Gamma (\alpha +\frac{1}{2},\beta +\frac{\beta_{\text{protected}}^{2}}{2\sigma^{2}})$
**Update** $\sigma^{2}$: $\sigma^{2}|\beta ,\tau^{2},Y∼\mathrm{IG}(\frac{n-1+p}{2},\frac{1}{2}(Y-X\beta {)}^{T}(Y-X\beta )+\frac{1}{2}\beta^{T}\text{diag}(\frac{1}{\tau^{2}})\beta )$
**Update** $\lambda^{2}$: $\lambda^{2}|\tau_{\text{non-protected}}^{2}∼\Gamma ((p-n_{\text{prot}})+a,\frac{1}{2}\sum_{\text{non-protected}}\tau_{j}^{2}+b)$

The Bayesian Lasso for dichotomous outcomes with partially protected variables uses the following Gibbs sampling sub-steps:

**Update** $\omega^{2}$: $\omega^{\left(t\right)}∼p(\omega \;|\;\beta^{\left(t-1\right)},\sigma ,\nu )$
**Update** *λ*: $\lambda^{\left(t\right)}∼p(\lambda \;|\;X,\beta^{\left(t-1\right)},\kappa )$
**Update** *z*: $z^{\left(t\right)}∼p(z\;|\;X,\beta^{\left(t-1\right)},\lambda^{\left(t\right)},\kappa )$
**Update** β: $\beta^{\left(t\right)}∼p(\beta \;|\;X,z^{\left(t\right)},\lambda^{\left(t\right)},\omega^{\left(t\right)},\nu ,\sigma ,\kappa )$
**Update** *ν*: $\nu^{\left(t\right)}∼p(\nu \;|\;\beta^{\left(t\right)},\sigma ,\kappa )$
**Calculate** the log posterior probability: ${\text{lpost}}^{\left(t\right)}=\log p(y,X,\beta^{\left(t\right)},\nu ,\kappa ,\sigma )$

Then after the last step the parameter values above at the iteration with the highest posterior probability are returned to complete a single iteration of the Gibbs sampling process.

### 2.1. Parameter specifications

Since some of the most common outcome variables specified in social science regression models are dichotomous, we modify the linear structure of the Partially-protected Lasso above for a logistic specification ( p(y=1 | X,β)=1/(1+exp⁡(−Xβ)), y∈{0,1}). However, this leads to interactable full conditional distributions for a Gibbs Sampler. Several authors have proposed limited simulation based estimation of Bayesian Logistic regression coefficients [11,12,17]. Our more general solution builds upon [11]'s simulation based regularized logistic regression by modifying the software reglogit by [11] to allow direct direct manipulation of the $\tau^{2}$ terms in a way that produces realizable full conditional distributions. The prior distribution on a single $\beta_{j}$ may be specified as per a Lasso or Ridge Regression (squaring the $\beta_{j}$ terms instead of taking the absolute values), controlled by a regularization parameter *ν* and scale parameter *σ*:

(5) $$\begin{array}{r} p(\beta_{j}\;|\;\sigma ,\nu )\propto \exp\left(-\frac{\nu }{\sigma }\sum_{i}|\beta_{j}|\right). \end{array}$$

The regularization of non-protected explanatory variables is affected by manipulating the *σ* term, where progressive increases cause the associated $\beta_{j}$ coefficients to decrease towards zero in the standard Lasso fashion as in the *λ* term in (1). Conversely the protected variables are not affected by the regularization process, so they remain invariant throughout this shrinkage process. Representing the set of *j* indices corresponding to the protected variables by P (protection), the simple algorithm is:

(6) $$\begin{array}{r} p(\beta_{j}\;|\;\sigma ,\nu )\propto \left\{ \begin{array}{ll} \exp\left(-\frac{\nu }{\sigma }|\beta_{j}|\right), & \text{if}\;j\notin P\\ 1, & \text{if}\;j\in P \end{array}\right. \end{array}$$

so that when j∈P this Lasso does not affect the distribution of $\beta_{j}$. See the appendix for computational details of this Gibbs Sampler. In the supplementary materials, we also provide source code for our package ProtectR to implement Bayesian Partially-Protected Regularization in linear models as well as an updated version of the [11]'s package reglogit for dichotomous variables.

### 2.2. Empirical evaluation

To substantiate the efficacy of the BPL approach we implemented it with two very different datasets from published work in political science that use the standard Lasso. Beforehand, we explore the properties of the BPL with simulation. We created a dataset of 1000 observations, with 20 predictor variables in X and a single outcome variable y. The true relationship between the predictor variables and the outcome variable is linear. The predictor variables are divided into two groups: the first group consists of five variables, which have a strong signal, while the second group includes the remaining 15 variables, which have a weak signal (i.e. more noise). The strong signal variables are generated by drawing random numbers from a standard normal distribution ( μ=0, σ=1), whereas the weak signal variables use a higher standard deviation ( μ=0, σ=10). The true coefficients for the predictor variables are set to 1 or -1 for the strong signal variables and 0 for the weak signal variables. This implies that the outcome variable is only affected by the strong signal variables. The outcome variable y is generated by taking the product of the *X* matrix and the true coefficients, and adding N(0,20) random noise.

**Figure 1.**
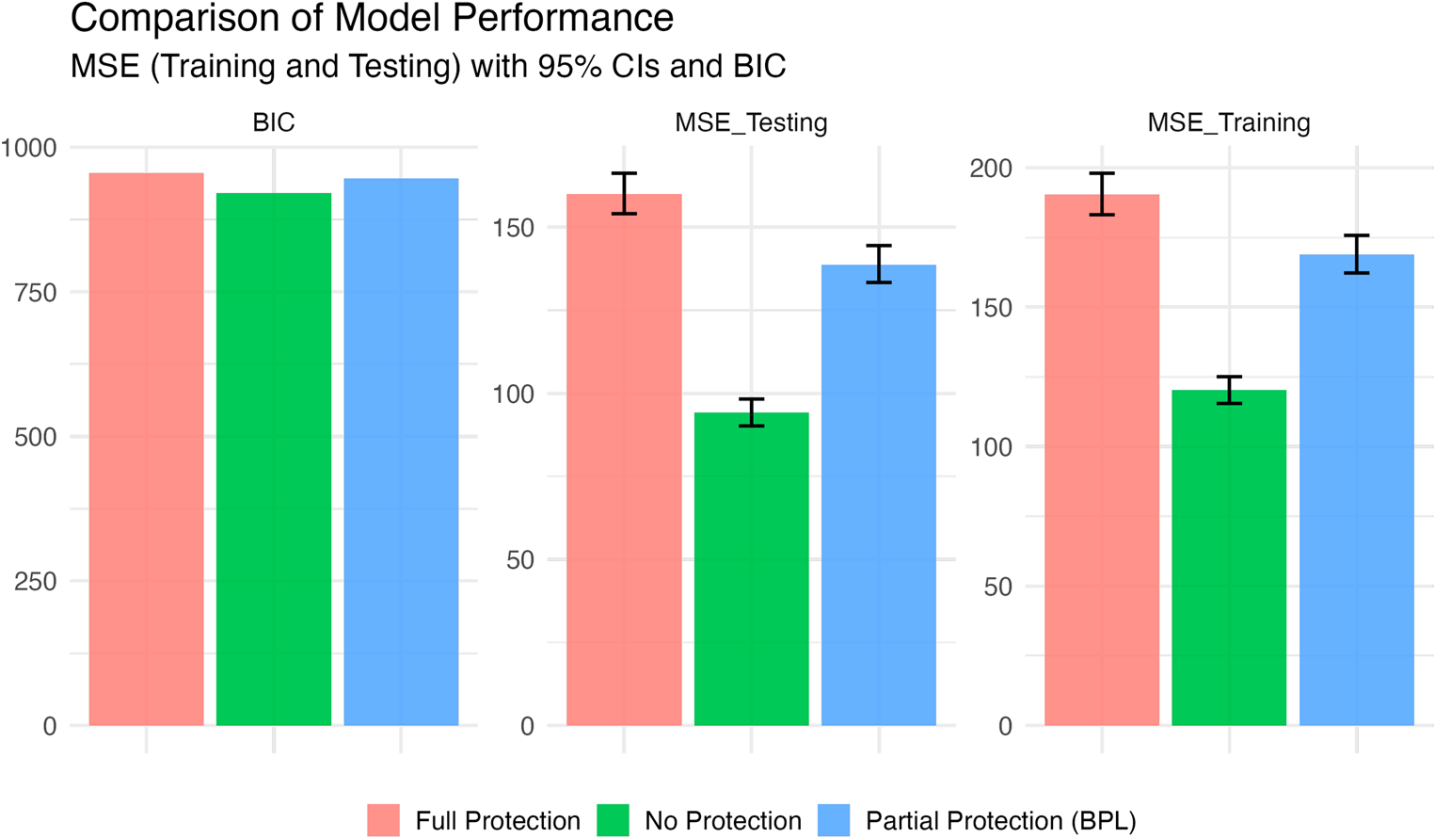
Prediction error comparison for simulated data.

There are three estimation processes for comparison with the simulated data: the standard Bayesian Lasso that allows any of the coefficients to shrink towards zero if it provides better fit (No Protection), a BPL protecting coefficients corresponding to explanatory variables with the stronger signal, and a standard Bayesian linear regression, all the predictors were protected (Full Protection). To assess the models' performance, we computed the Mean Squared Error (MSE) and Bayesian Information Criterion (BIC) for the training and testing datasets, with lower values signifying better accuracy. BPL, which selectively protected variables, outperformed the full protection model in terms of MSE but fell slightly short of the standard Bayesian Lasso. This trade-off reflects the cost of safeguarding theoretically driven variables. As illustrated in Figure 1, is a comparison using the Bayesian Information Criterion (BIC), a measure that considers both fit and complexity: −2 times the fitted likelihood + *p* times the log of *n*. Here again, BPL demonstrated superior performance compared to standard Bayesian linear regression, with lower BIC values indicative of a better model.

**Figure 2.**
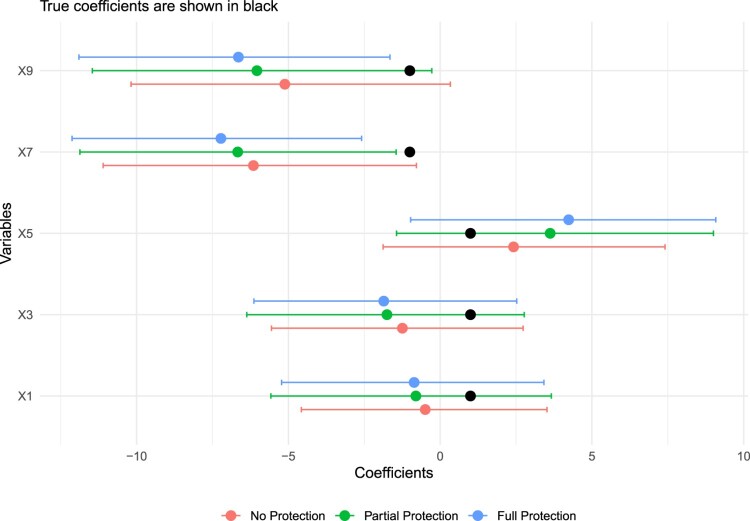
Comparison of coefficients for theoretically driven (and strong-signal) variables across models using simulated data.

**Figure 3.**
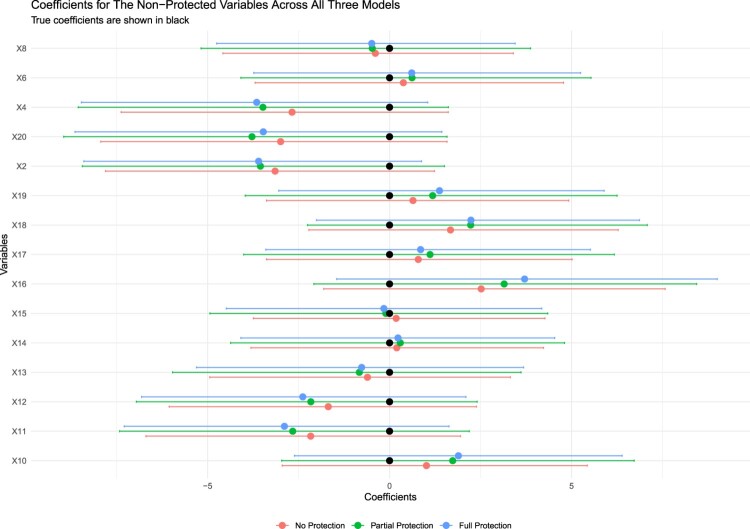
Comparison of coefficients for weak-signal variables across models using simulated data.

When checking the extent of coefficient shrinkage for strong-signal variables in Figure 2, we observe that our method allows partial-protection for the protected variables. In other words, it is not that they are excluded from the regression since they still help inform other coefficients in the Gibbs Sampler. Hence, their coefficients are almost always between the fully protected model and no protected model. More importantly, when there are theoretically driven but small-effect variables, like X9 in our case, they can be easily shrunk to zero in a regular Bayesian Lasso. Figure 2 shows that protecting this variable saved it from shrinkage, and it also managed to cover the true value within the associated credible interval. Another issue we want to note is that the BPL does not cause an *unnecessary* protection, or save some non-theoretically variables from shrinkage by mistake. This could be seen in Figure 3, where all variables' true coefficients are 0. With all models, including the BPL, the 95% credible intervals contain the true coefficient at 0.

Next we reanalyze two different datasets from very recent published work that use a conventional Lasso, one with an interval measured outcome and the other with a dichotomous outcome. Neither uses very large data (*n* = 601 training and *n* = 254 testing in the first example, and *n* = 1556 training and *n* = 667 testing in the second example) where the differences in estimation procedure would be pronounced, but there are important observable differences in the quality of the results. The reanalysis and extensions of existing models provide considerable insight into the performance and implications of protected Bayesian regularization.

*Revisiting forced migration: A machine learning perspective *[19]**. This study applies random forest and Lasso to analyze the determinants of forced migration in 45 African countries from 1997 to 2017. The outcome variables under consideration are refugee flows and asylum applications, derived from the UNHCR Population Statistics online database and the Eurostat database. The predictors include a wide range of factors such as internal political conflict (based on ACLED data), real GDP per capita, internet usage, net official development assistance and aid received, political regime and institutional characteristics, as well as environmental determinants like natural disasters, precipitation, and temperature anomalies. In the context of this study, theoretically driven protected variables include *key indicators of political terror and GDP per capita*, which are seen as critical driving forces of forced migration. The mean square error (MSE) values are 7,418,180 for no protection, 7,467,819 for partial protection, and 7,675,015 for full protection. This demonstrates that there is a fit penalty for mandating all of the variables. This underscores the potential trade-off between preserving the significance of theoretically important variables and maintaining optimal model accuracy, emphasizing the need for a judicious application of protected Bayesian regularization.

*Predicting politicians' misconduct: Evidence from Colombia *[9]**. The article applies machine learning models, including Lasso, to predict mayoral corruption at the municipality level in Colombia, using 147 municipality characteristics as predictors. The outcome variable is mayoral corruption, while predictors span ten dimensions, including public sector attributes, human capital, economic activity, and others. Lasso is used for its regularization properties, which promote model parsimony and reduce overfitting. The theoretically-driven protected variables are *judicial offices in each municipality*. As we believe the number of judiciaries and judiciary offices in each municipality is directly linked to the corruption levels, we protected those variables from shrinkage in the Partially-protected models. The area under the curve (AUC) values are 0.716 for no protection, 0.717 for partial protection, and 0.665 for full protection. So in this case partial protection produced a slightly better overall fit, but this is well-within rounding and Monte Carlo error differences so they are essentially the same. Recall that an unencumbered regular Lasso will find the best set of predictions, but it is not guaranteed to fit better by other metrics. Interestingly here, full protection in this case gives a noticeably worse overall fit showing a strong protection penalty.

In this study, we introduce the Bayesian Partially-Protected Lasso (BPL), a novel approach designed to balance the dual imperatives of rigorous statistical analysis and respect for theoretical constructs. The BPL acknowledges that not all variables warrant equal treatment in regression models, and therefore, allows researchers to selectively protect variables grounded in strong theoretical principles. To facilitate its application in real-world scenarios, we have developed an R package ProtectR. This user-friendly tool streamlines the implementation process, enabling researchers to integrate the BPL seamlessly into their analytical workflow, significantly simplifying what could otherwise be a complex undertaking.

The potential advantages for empirical social science are manifold. First, the BPL method provides a means to preserve the integrity of theoretical constructs without sacrificing the power and adaptability of regression models. By doing so, it bridges the often-discussed divide between theory-driven and data-driven research, fostering a more harmonious integration of these two vital pillars of scientific inquiry. The Bayesian Partially-Protected Lasso (BPL) approach offers a structured method to recognize important covariates ex ante, potentially assisting in retaining theoretically informed variables within the model. Particularly in situations of high multicollinearity, where predictors might exhibit overlapping influences, conventional methods can sometimes unduly diminish or overlook certain essential variables. BPL, in its design, seeks to give precedence to ‘theoretically motivated’ variables, aiming to reduce the risk of arbitrary shrinkage. Furthermore, the introduction of our R package aids in democratizing this method. By lowering the technical barriers to entry, even those not deeply entrenched in the nuances of machine learning tools can harness the power of BPL. This inclusivity ensures a broader reach and application across subfields, ultimately enriching the academic discourse and facilitating the generation of more nuanced, theory-aligned model specifications.

In the ever-evolving landscape of social science, particularly in disciplines like political science, there is an increasing trend towards harnessing the capabilities of machine learning tools primarily for predictive purposes. However, the overarching emphasis on prediction, often at the expense of robust theoretical foundations, can lead to a narrow understanding and potentially skewed interpretations of complex socio-political phenomena. The BPL is a new step in the direction of bridging this gap, aiming to integrate theory intricately into predictive modeling. The BPL serves as a reminder that theory and prediction are not mutually exclusive but can coexist synergistically. For the future of political science research, and social sciences more broadly, it is important to have an active pursuit of strategies that thoughtfully blend machine learning's predictive prowess with the depth and richness of theoretical constructs.

## 3. Bayesian elastic net

We now pivot to an extension of the just described technology. Most literature in the social sciences includes a collection of explanatory phenomena that need inclusion because the supporting theories are very strong. Often, the decision involves selecting which measured version of the phenomenon should serve as a right-hand side variable. Ref. [16] referred to these as ‘inside the horizon’ variables, emphasizing their established value. In a voting choice model, these variables include income, partisanship, ideology, sex, race, age, region, family status, and education. According to Leamer, the challenge lies in specifying an additional set of ‘over the horizon’ variables that may provide new knowledge. Researchers often include the first type of variables in the final specification even if they are not found to be statistically reliable, due to a history of contributing to model specifications in the relevant literature.

A fundamental conflict exists in modern social science regression modeling (‘regression’ in the broadest sense): all associated fields increasingly generate *gigantic* datasets, but social scientists have deeply entrenched and important phenomena that must be used as historically reliable explainers. The problem of the 20th century was often the lack of explanatory variables, but now there are actually *too many* possible explanatory variables in the ‘data century’ [10]. Moreover, this disconnect will likely intensify as new datasets from various sources like IoT, internet traffic, digital video, genomics, and more, enter the analysis realm. We address this salient challenge: how do you deal with the proliferation of useful but very large datasets while preserving fealty to a long and rich social science literature?

Addressing this gap, we introduce the Bayesian Protected Elastic Net (BPEN), an innovative approach that extends the conventional Elastic Net model within a Bayesian framework to incorporate protections for variables deemed theoretically significant. Our approach is also an extension of the recently introduced Bayesian Protected Lasso (BPL) described above. By integrating a mechanism for selective shrinkage, the BPEN allows researchers to safeguard key variables from penalization, thereby honoring theoretical commitments while leveraging the benefits of regularization to contend with high-dimensional data. This methodological advancement represents a synthesis of predictive efficiency and theoretical conscientiousness, tailored to the nuanced demands of social science research. As far as we are aware, no research has been done to study the effect of using differential penalization, either in a frequentist or Bayesian sense.

We provide the mathematical underpinnings of the BPEN, detailing its formulation and the statistical principles that facilitate the differential treatment of protected versus non-protected variables. The introduction of variable-specific priors enables the flexible calibration of shrinkage, allowing the BPEN to adaptively refine model complexity without compromising the integrity of theoretically indispensable variables.

To empirically validate the BPEN's efficacy, we conduct a comprehensive evaluation using a simulated dataset designed to closely mimic the challenges encountered in high-dimensional social science research, and a real-world dataset on conflict prediction. The simulation is meticulously designed to mimic the complexities of real-world social science data, enabling a rigorous assessment of the BPEN's performance relative to existing methods. The empirical applications serve as a critical proving ground, demonstrating the BPEN's capability to maintain model accuracy and interpretability while steadfastly preserving theoretical variables.

This paper has two important and unique methodological contributions that do not currently exist in the statistical literature or elsewhere. *First*, it extends a novel methodological tool developed for Lasso to the Elastic Net, offering a sophisticated solution to the problems posed by high-dimensional data that is now prevalent. *Second*, it balances the computational strengths of machine learning with the rich theoretical traditions of social sciences, thus balancing two competing criteria while demonstrating methodological pluralism. Through the Bayesian Protected Elastic Net, we provide a bridge over the divide between large-scale data-driven discovery and theory-driven inquiry, which is now a very common problem in many scientific literatures.

## 4. The standard elastic net

In the context of the standard linear model, consider X as the n×p matrix of standardized predictors, which includes a leading column of ones for the intercept, y as the *n*-dimensional outcome variable vector, β as the *p*-dimensional vector of regression coefficients, and $\sigma^{2}$ as the residual variance. To address overfitting and variable selection in high-dimensional data where the number of predictors may exceed the number of observations, the Elastic Net combines the penalties of the Lasso and Ridge regression as follows [31]:

(7) $$\begin{array}{r} {\hat{\beta }}_{\text{EN}}=\arg\min_{\beta }\left[{\left(\tilde{y}-\mathrm{X\beta}\right)}^{\prime }\left(\tilde{y}-\mathrm{X\beta}\right)+\lambda_{1}\sum_{j=1}^{p}|\beta_{j}|+\lambda_{2}\sum_{j=1}^{p}|\beta_{j}{|}^{2}\right]. \end{array}$$

where $\lambda_{1}$ and $\lambda_{2}$ are penalty terms that control the extent of Lasso and Ridge regularization, respectively. The Elastic Net is particularly effective in scenarios with highly correlated predictors, as it allows for the grouping effect where correlated predictors are either included or excluded from the model together. There are also extensions of the elastic net such as the *restricted bridge estimator* (BRIDGE) of [29] where the penalty function is simply $\sum_{j=1}^{p}|\beta_{j}{|}^{q}$, and the special cases of the Lasso, Ridge, and Elastic Net follow.

The determination of the penalization parameters $\lambda_{1}$ and $\lambda_{2}$ is crucial in moving the model towards Lasso or Ridge penalization, and can be achieved through expert judgment or algorithmically via cross-validation. In the standard, non-Bayesian Elastic Net scenario, if we wanted to protect some variables from shrinkage, we could have assigned an additional weight *w* parameter to the penalization terms $\lambda_{1}$ and $\lambda_{2}$. Under such an arrangement, the model's objective function would be adjusted to incorporate these weights, effectively modifying the balance between Lasso and Ridge penalties for each variable based on the specified weights:

(8) $$\begin{array}{r} {\hat{\beta }}_{\text{EN(w)}}=\arg\min_{\beta }\left[{\left(y-\mathrm{X\beta}\right)}^{\prime }\left(y-\mathrm{X\beta}\right)+\lambda_{1}\sum_{j=1}^{p}w_{j}|\beta_{j}|+\lambda_{2}\sum_{j=1}^{p}w_{j}|\beta_{j}{|}^{2}\right]. \end{array}$$

The vector $w=(w_{1},w_{2},\ldots ,w_{p})$ specifies individual weights for each of the *p* coefficients, facilitating differential penalization across the model. A higher weight $w_{j}$ implies greater penalization for the *j*th coefficient, effectively encouraging more significant shrinkage. Conversely, a lower $w_{j}$ offers a degree of protection against shrinkage for the associated coefficient, allowing it to remain more influential in the model despite the regularization process.

Despite its advantages, the standard Elastic Net framework as described has limitations. First, non-Bayesian models generally lack a mechanism for directly incorporating prior knowledge or theoretical considerations into the modeling, potentially leading to the exclusion of variables critical to the substantive research. Theoretically, one could use the *λ* or *w* values as a way to incorporate prior information into the model, but shrinkage parameters have not traditionally been used or tested in this manner. Thus, we do not have a *reliable* method of integrating information from previous literature into the model with traditional regularization methods.

Another fundamental drawback of standard penalization methods, including both the Lasso and Elastic Net, is their inability to provide reliable standard errors for the coefficients. This shortcoming significantly hampers the inferential statistics necessary for understanding the precision of parameter estimates. Without reliable standard errors, the quantification of uncertainty around coefficient estimates becomes challenging, undermining the capacity to make statistically grounded inferences about the importance of predictors. This limitation is especially severe in social science research, where the interpretation of effect sizes and their confidence intervals are integral to substantiating theoretical claims.

## 5. Bayesian partially-protected elastic net

Due to these limitations, penalized regression methods have been effectively extended into the Bayesian framework through the introduction of specific prior specifications and the application of modern simulation techniques for estimation. Early pioneers in the development of Bayesian penalization techniques demonstrated that these methods could achieve comparable, if not superior, results in minimizing prediction error [7,13,15,18,24,26]. Beyond the promise of reduced prediction error, Bayesian penalization offers several distinct advantages: it seamlessly integrates the penalty term into the prior structure, facilitates the derivation of valid standard errors and parameter estimates from the posterior distributions, and enables the inclusion of additional parameters in prior specification. The ease of prior specification plays a critical role by enabling the selective protection of certain variables from shrinkage, particularly when theoretical considerations underscore their importance. Previous seminal works compellingly advocated the efficacy and advantages of Bayesian regularization, notably elaborating on the adoption of a double exponential (Laplace) prior for coefficient shrinkage [15,22].

Another salient characteristic that sets Bayesian regularization apart from traditional methods is its flexible approach to coefficient shrinkage. Unlike conventional methods that might shrink coefficients to exactly zero, Bayesian penalization methods may or may not exactly shrink coefficients to zero. However, since it provides reliable credible intervals for the parameter estimates, researchers have developed nuanced strategies to select variables after shrinkage [21]. Scholars proposed various methods such as the scaled neighborhood criterion [18], a predetermined threshold value [8], and the credibility interval criterion [26]. Particularly, the credible interval criterion posits that a coefficient is considered effectively zero if its credible interval encompasses zero. This methodological flexibility is not viewed as a drawback but rather as a strength of the Bayesian approach. It allows for the identification of non-reliable coefficients in a regression context with the added advantage of providing them with a systematically derived standard error, an attribute not available in traditional non-Bayesian techniques.

Consider X as the matrix of standardized predictors, y as the outcome variable, β as the vector of regression coefficients, $\sigma^{2}$ as the residual variance, $\lambda_{1}$ and $\lambda_{2}$ as the hyperparameters for the penalty terms corresponding to $L_{1}$ and $L_{2}$ regularization, respectively, and $\tau^{2}$ representing the precision of coefficients. Let *n* denote the number of observations, *p* the total number of predictors, and $p_{\text{prot}}$ the count of protected variables. The Bayesian Elastic Net model, accommodating protected variables, is conceptualized as follows:

(9) $$\begin{array}{rl} y & =\alpha +X\beta +\epsilon ,\\ \epsilon_{i} & ∼N(0,\sigma^{2}),\;i=1,\ldots ,n,\\ \beta_{j} & ∼\left\{ \begin{array}{ll} \text{Laplace}(0,\lambda_{1}/\sigma )\times \text{exp}\left(-\frac{\lambda_{2}}{2}\beta_{j}^{2}\right), & \text{for regularized coefficients},\\ N(0,10^{4}), & \text{for protected coefficients}, \end{array}\right.\;j=1,\ldots ,p. \end{array}$$

where the variance parameter in the last normal expression is arbitrary and diffuse but shows little sensitivity. We chose to use a highly diffuse prior on the protected variables by specifying a large variance because we wanted to make the data for these variables ‘work harder’ to produce a posterior distribution with good properties.

The priors in Bayesian Elastic Net are tailored to embody the Elastic Net's balancing act between the Lasso's sparsity-inducing L1 penalty and the Ridge regression's variance-controlling L2 penalty. However, rather than employing a singular mixed Gaussian model for all coefficients, our implementation distinguishes between coefficients based on their theoretical importance or relevance to the research question at hand. For coefficients identified for regularization, we apply a *Laplace prior*–characteristic of the Lasso for its sparsity-inducing properties–alongside a *Gaussian penalty* reminiscent of the Ridge, directly within the model's likelihood function. This dual approach allows for the nuanced application of both penalties, where the Laplace prior aggressively pushes coefficients towards zero, and the Gaussian term smooths the estimates by penalizing their squared values, effectively achieving the Elastic Net's characteristic regularization. Formally speaking, for each coefficient targeted for regularization, denoted by $\beta_{\text{reg}}$ for predictors where a binary indicator $I_{\text{reg}}[j]=1$, we apply a Laplace prior to impose the Lasso's L1 penalty and directly integrate an L2 penalty into the posterior log-likelihood for the Ridge effect. Mathematically, this dual penalization is represented as follows:

(10) $$\begin{array}{r} \beta_{\text{reg}}[j]∼\text{Laplace}(0,\lambda_{1})\;\text{for}\;I_{\text{reg}}[j]=1, \end{array}$$

indicating the L1 penalty via a Laplace distribution with parameter $\lambda_{1}$. Then we incorporate the L2 penalty by adjusting the model's target log posterior:

(11) $$\begin{array}{r} \text{Target log posterior adjustment for L2:}-\frac{\lambda_{2}}{2}\beta_{\text{reg}}[j{]}^{2}, \end{array}$$

where $\lambda_{2}$ moderates the extent of shrinkage towards zero, embodying the Ridge penalty. This formulation ensures that coefficients under regularization are influenced by both sparsity-inducing and shrinkage effects, corresponding to the Elastic Net's objectives. For predictors protected from regularization, denoted by $\beta_{\text{prot}}[j]$ where $I_{\text{reg}}[j]=0$, we specify non-informative priors to avoid influencing their estimates unduly:

(12) $$\begin{array}{r} \beta_{\text{prot}}[j]∼N(0,10^{4}), \end{array}$$

reflecting a broad normal distribution that imposes minimal a priori constraints on $\beta_{\text{prot}}[j]$ values.

Given the priors, when calculating the posterior distributions, hence, we differentiate the coefficients coefficients for protected variables ( $\beta_{\text{prot}}$) and those for non-protected variables ( $\beta_{\text{reg}}$).The posterior distribution that incorporates this distinction is given by:

(13) $$\begin{array}{rl} & p(\beta_{\text{reg}},\beta_{\text{prot}},\alpha ,\sigma^{2}\;|\;y,X,\lambda_{1},\lambda_{2})\\ & \;\times \propto \underset{\text{Likelihood}}{\underbrace{p(y\;|\;X,\beta_{\text{reg},\beta_{\text{prot}},\alpha ,\sigma^{2})}}}\underset{\text{Priors}}{\underbrace{p(\beta_{\text{reg}}\;|\;\lambda_{1},\lambda_{2},\sigma^{2})p(\beta_{\text{prot}})p(\alpha )p(\sigma^{2})}}, \end{array}$$

where:

-
$p(Y\;|\;X,\beta_{\text{reg}},\beta_{\text{prot}},\alpha ,\sigma^{2})$ is the likelihood of the observed data given the model parameters.
-
$p(\beta_{\text{reg}}\;|\;\lambda_{1},\lambda_{2},\sigma^{2})$ denotes the combined prior for the regularized coefficients, incorporating both L1 (Laplace) and L2 (Gaussian) penalties.
-
$p(\beta_{\text{prot}})$ signifies the prior for the protected coefficients, which could be a non-informative or wide prior, indicating minimal regularization to retain their theoretical significance.
-
p(α) and $p(\sigma^{2})$ are the priors for the intercept and error variance, respectively.

Then, to identify the optimal L1 ( $\lambda_{1}$) and L2 ( $\lambda_{2}$) penalization terms for our Bayesian Protected Elastic Net model, we implement a *5-fold cross-validation strategy*. This method involves partitioning the data into five folds, systematically using four folds for training and one fold for validation, iteratively across a randomly created grid of $\lambda_{1}$ and $\lambda_{2}$ values. We then determine the optimal combination of these penalization terms by finding the pair that minimizes the Mean Squared Error (MSE) across the validation folds. This approach ensures that the selection of $\lambda_{1}$ and $\lambda_{2}$ is both empirically grounded and robust against overfitting.

This nuanced implementation not only aligns with the Bayesian Elastic Net's theoretical underpinnings but also enhances its practical utility by providing a mechanism to incorporate domain-specific knowledge and theoretical considerations into the regularization process. By doing so, it addresses a critical gap in traditional penalization methods, which, despite their predictive prowess, often fall short in terms of interpretability and adherence to substantive theoretical frameworks.

### 5.1. Empirical evaluations of BPEN

To empirically test our proposed method, we applied BPEN on two datasets: The first is a simulated dataset, and the second one is a real-world scenario on conflict prediction.

#### 5.1.1. Simulation study

For this simulation we first generated a dataset which comprises *n* = 100 observations and *p* = 20 predictor variables, denoted as $x_{1},x_{2},\ldots ,x_{20}$, alongside a response variable *y*. The relationship between the predictors and the response is modeled linearly, with predictors divided into two categories based on the strength of their signal:

- *Strong Signal Variables*: $X_{1}$, $X_{3}$, $X_{5}$, $X_{7}$, and $X_{9}$ are generated from a standard normal distribution N(0,1), indicating their significant influence on the outcome variable *y*.
- *Weak Signal Variables*: The remaining 15 predictors are sampled from a normal distribution with a higher variance N(0,10), representing variables with negligible impact on *y* due to increased noise.

The true coefficients $\beta_{j}$ associated with each predictor variable *j* are determined as follows:

$$\beta_{j}=\left\{ \begin{array}{ll} 1 & \text{if}\;j\in \{X_{1},X_{3},X_{5}\},\\ -1 & \text{if}\;j\in \{X_{7},X_{9}\},\\ 0 & \text{otherwise}. \end{array}\right.$$

where coefficients of ±1 signify strong signals, influencing *y* positively or negatively, and a coefficient of 0 indicates a lack of direct effect.

The outcome variable *y* is constructed using the linear model Y=Xβ+ϵ, where *ϵ* represents normally distributed noise N(0,20), adding an additional layer of complexity to the simulated data. This setup allows us to assess the performance of our method in distinguishing between and appropriately handling variables of differing theoretical and empirical significance.

Using the simulated dataset, we model the relationship between the predictors and the outcome variable through the implementation of three distinct models using Stan, although other estimation approaches are reasonable as well [5,14,20,23,27,28,30]. The first model is the standard Bayesian Elastic Net, in which we protect no variable, so all variables are subject to the same regularization. The second model is our proposed Bayesian Partially Protected Elastic Net (BPEN). Here, we protect some variables; we select these protected variables based on their established importance in previous literature, particularly those consistently shown to affect the outcome variable. Finally, we apply standard Bayesian linear regression, where we protect all variables–thus, no variable is subject to any penalization (Full Protection). Importantly, in all models, all variables are included in the regression model.

**Figure 4.**
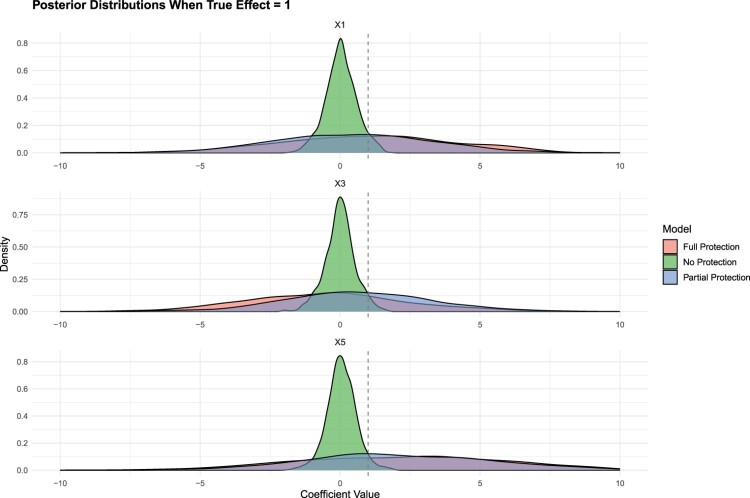
Prediction error for the simulated data.

To assess the efficacy of the aforementioned models, we used the Mean Squared Error (MSE) metric on both training and testing datasets as a measure of performance. A lower MSE value signifies enhanced model accuracy by denoting reduced discrepancies between predicted and observed outcomes. Our findings for the simulation in Figure 4 reveal that BPEN, which safeguards variables identified to possess a stronger theoretical signal, outperforms the fully protected model in terms of MSE. However, it marginally lags behind the standard Bayesian Elastic Net (no protection). This trade-off underscores the methodological cost associated with the intentional preservation of variables deemed significant from a theoretical perspective. The reason why the training and testing MSEs are close to each other is the way we train the models: Instead of using the whole training dataset, we employed 5-fold cross-validation within the training set. Hence, we do not observe a large difference between training MSE and testing MSE. In reality, for the partial protection and full protection models, the MSEs for the training set are even larger than their testing counterparts.

**Figure 5.**
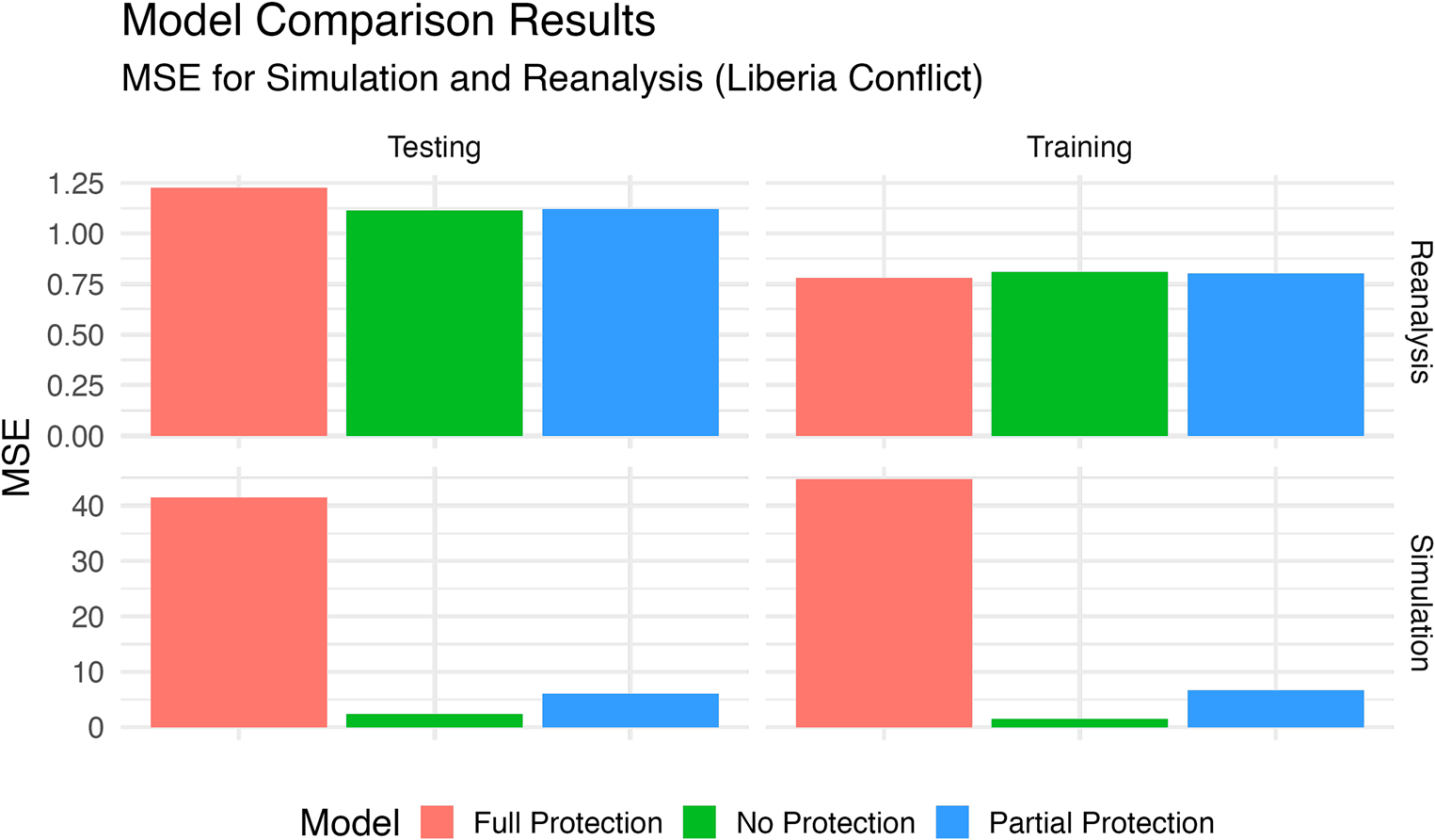
MSE comparison results for simulation and reanalysis.

Figure 5 presents the estimated coefficients for the cases where the true $\beta_{j}$ is 1 in the simulation. It is evident that the model without any protection–the one applying the Bayesian Elastic Net uniformly–performs suboptimally in capturing the true effect, with a tendency to shrink significant variables towards zero even though the real effect is higher than 0. Although all models technically capture the true effect within their credible intervals, the models with full and partial protection provide a more accurate estimate of the true effect, thanks to their less restrictive priors.

**Figure 6.**
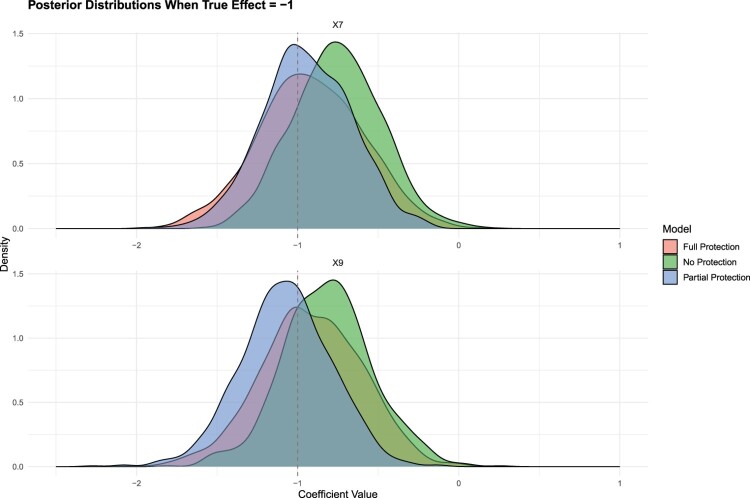
Posterior distributions when True\ Effect=−1.

In Figure 6, we turn our attention to instances where the true effect ( $\beta_{j}$) is −1. All models successfully identify the true effect for both variables; however, the models with full and partial protection demonstrate superior performance over the standard Bayesian Elastic Net.

**Figure 7.**
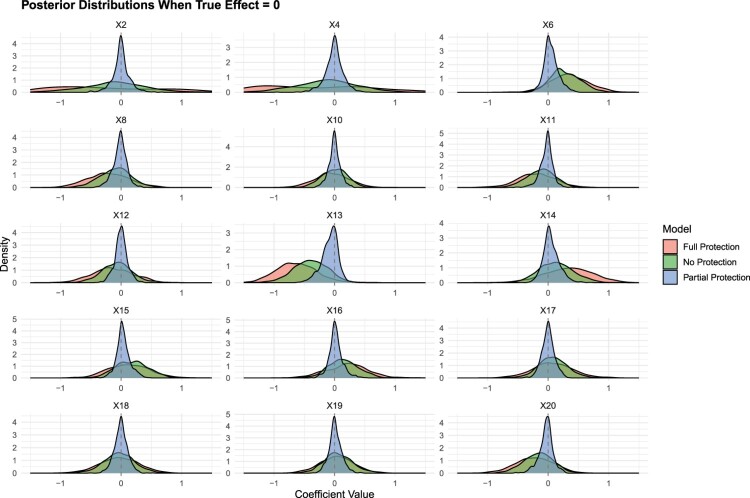
Posterior distributions when True Effect=0.

Finally, the scenario depicted in Figure 7 involves the 15 predictors for which there is no real impact of ( $x_{j}$) on the outcome variable *y*. The partially protected model (BPEN) excels at identifying the absence of an effect, surpassing both the fully protected and non-protected models in precision. This is particularly noticeable for variables $x_{2}$ and $x_{4}$, where the BPEN model yields considerably more concentrated posterior distributions, in stark contrast to the other models that produce broader distributions despite the true effect being zero. In other words, our model does not only do a good job when it comes to protecting theoretically motivated variables and capturing their effects more accurately, it also shrinks more when the variables are *not* theoretically motivated.

#### 5.1.2. Political violence in Liberia application

We now turn to real-world data in political science as a means of testing the Bayesian Penalized Elastic Net (BPEN) against a published application of the regular Lasso in the *Journal of Peace Research*. This is an important test for our new method since it uses data from a published application of the obvious alternative. In their study on local violence prediction, Ref. [6] used a Lasso to predict local political violence in Liberia from a dataset with 56 original variables. Their objective was to reduce this dimension space of potential risk factors to a core set that can be easily interpreted from a policy perspective. Their application of the Lasso reduced the number of potential covariates down to only five with strong predictive power. We also picked this study as a straw-man for comparison because these authors have already found in [6] that their Lasso approach outperforms other machine learning alternatives such as random forests, neural networks, and the standard logistic regression model. This allows us to very compactly compare the BPEN against a wide range of approaches for this type of data. This re-analysis of the political violence data also shows that regularization tools are able to isolate key predictors of political violence in this dataset, such as ethnic heterogeneity and polarization, while disregarding less pertinent variables.

The re-analysis here specifies three distinct regularization algorithms applied to the political violence data using a Bayesian linear model: (1) a ‘No Protection’ Elastic Net, (2) our ‘Partially Protected’ version of the Elastic Net developed in this work, and (3) a ‘Full Protection’ specification with no regularization at all using the variables that Ref. [6] identified with their original standard Lasso: Exposure to War Violence, Frequency of Police/NGO Visits, Percentage of Contribution to Public Facilities, Perception of Violence by Other Tribes, Minority Tribe Presence in Town Leadership, and the Commodity Price Index. The comparative question is which of these approaches is more accurate in predicting violent occurrences in 2012 by incorporating these theoretically informed predictors.

**Figure 8.**
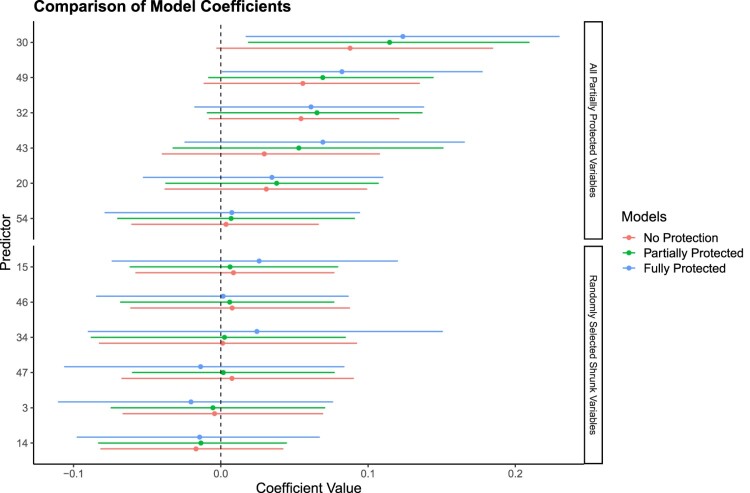
Coefficient comparison for various models when predicting local conflict in Liberia. The first 6 variables on the *y* axis is the ones we protected in partially protected Elastic Net model. The second set of predictors on the *y* axis are the ones that we allowed BPEN to shrink. The plotted points represent the posterior median values, and the error bars correspond to their 95 percent credible intervals.

All three regularization approaches are implemented in R for these data and the resulting highest posterior density intervals are shown in Figure 8. It is clear that the BPEN moderates the shrinkage of most variables, particularly for the initially protected set. However, the ‘No Protection’ Elastic Net, which indiscriminately targets variables even if theoretically important, shrinks the partially protected set from the BPEN more towards zero. This is exactly as predicted by our theoretical discussion and the discussion of how the Markov chain Monte Carlo process works. Notably, variable V30 has an estimator in Figure 8 that exemplifies the efficacy of our approach. The BPEN effectively preserves this predictor's coefficient from shrinkage, acknowledging its theoretical relevance, while the indiscriminate shrinkage of the non-protected Elastic Net shrinks it sufficiently that the highest posterior density interval now covers zero.

Now consider the necessary trade-off in model performance for these three regularization approaches. Meaning, what is the price that has to be paid to protect a set of theoretically important variables in the investigation of political violence in Liberia? In the second half of Figure 4 we provide the mean square error (MSE) in training and testing scenarios for the three alternatives *since this the appropriate accuracy measure because we are specifying linear models* (as opposed to posterior predictions, which would require setting an arbitrary interval on the real line like >0, etc). As anticipated by the theoretical discussion, the ‘No Protection’ approach gives the lowest MSE value at 1.115, but of course, it does not protect any theoretically important variables that a political researcher might find necessary. The cost of partial protection modest in MSE terms: 1.121−1.115 = 0.006. However, it is up to the individual researcher to decide whether this is an acceptable level. Finally, note that the ‘Full Protection’ approach with no allowed shrinkage gives a higher MSE cost over the BPEN, 1.227−1.121 = 0.106, than we paid for using the BPEN over the unprotected Elastic Net. These findings demonstrate the efficacy of BPEN in integrating theoretically informed predictors from extant literature with minimal compromise on model accuracy.

#### 5.1.3. High dimensional data: ANES

Finally, to evaluate our model, we applied it to the 2020 American National Election Survey (ANES) dataset–a high-dimensional data source that is ideal for illustrating the strengths of Elastic Net Regularization. In doing so, we mirrored the methodology of [30], who employed a protected Bayesian Lasso on the ANES data by safeguarding a set of theoretically important variables. Specifically, we adopted the same protection strategy for key predictors (such as age, education, church attendance, race, gender, income, ideology, and evangelical status) during the regularization process.

The goal of [30] was to predict feeling thermometer ratings for presidential candidates while preventing theoretically critical variables from being shrunk to zero. Their approach was designed to reconcile traditional research–which emphasizes the inclusion of conceptually important predictors–with modern, prediction-focused methods that often overlook the theoretical importance of individual variables.

For our analysis, we closely followed their data preparation steps. We retained only pre-election variables, addressed missing data using multiple imputation via the MICE package in R, and removed variables with excessive missing values or near-zero variance. The resulting dataset consisted of 385 variables across 6843 observations. In line with [30], we divided the dataset into training (70%) and testing (30%) subsets, with Joe Biden's feeling thermometer scores serving as the outcome variable.

Using this framework, [30] demonstrated the advantages of protecting theoretically important variables, showing that their method either preserved or improved model performance compared to unprotected approaches. By replicating their variable selection process and analytical steps, we aim to highlight the comparative properties of the Elastic Net model in high-dimensional settings.

**Figure 9.**
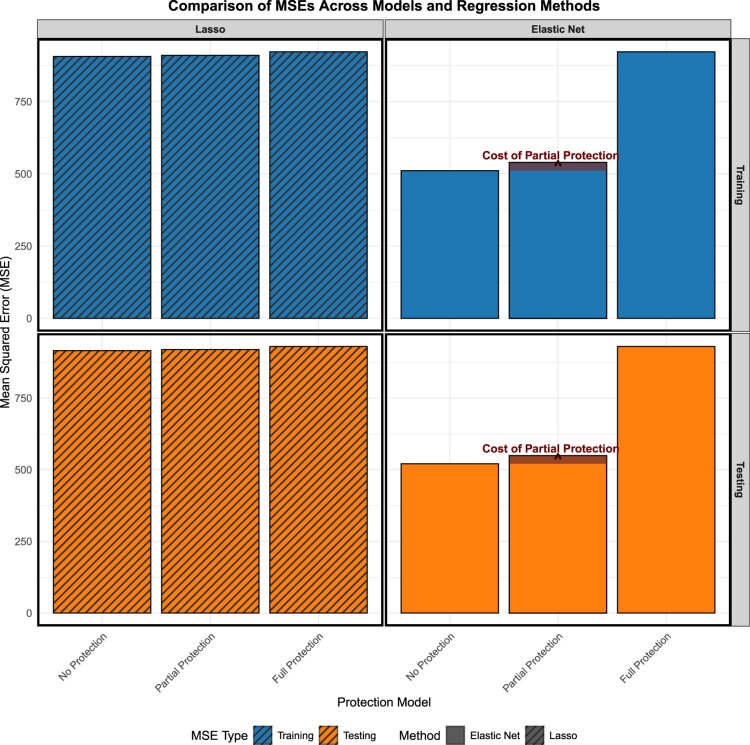
Comparison of MSE scores for BPL [30] and BPEN.

Figure 9 compares the performance of different protection strategies within the Bayesian Elastic Net framework, building on the insights of [30]. The figure illustrates how model protection choices affect mean squared error (MSE) and highlights the cost of partial protection in the Bayesian Partially-Protected Elastic Net (BPEN).

For the Elastic Net model, the No Protection strategy achieved the lowest MSEs on both the training (510.75) and testing (520.55) sets, as allowing unrestricted shrinkage enables the model to fine-tune coefficients aggressively for error reduction. In the Partial Protection setting–where certain theoretically important variables are shielded from shrinkage–the MSEs were slightly higher (539.58 for training and 549.39 for testing), reflecting a modest increase in error in exchange for preserving key predictors. In contrast, the Full Protection strategy, which prevents any shrinkage across all variables, resulted in the highest MSEs (922.42 for training and 929.74 for testing), indicating that overly restricting the model significantly degrades predictive performance.

A similar trend was observed in the results reported by [30] under the Lasso framework. There, the No Protection model produced the lowest MSEs (906.34 for training and 915.31 for testing), followed by the Partial Protection model (910.07 for training and 918.98 for testing), and finally the Full Protection model with the highest errors (922.42 for training and 929.74 for testing).

These findings demonstrate that while No Protection maximizes predictive accuracy, it overlooks the theoretical significance of certain predictors. Conversely, Full Protection prioritizes theory at the cost of substantial predictive performance loss. Partial Protection offers a balanced compromise–only a moderate increase in MSE relative to the unrestricted model, while still safeguarding key variables–making it a practical choice in high-dimensional settings where both prediction and interpretability are valued.

## 6. Conclusion

This study has put forth the Bayesian Partially Protected Elastic Net (BPEN) as a methodological innovation that adeptly negotiates the delicate balance between theoretical prudence and predictive precision. Through rigorous simulations, we have demonstrated that the BPEN not only preserves the theoretical underpinnings of key variables but also either protects or enhances the model's predictive fidelity. Our approach, therefore, does not simply cater to the dichotomy of predictive versus explanatory modeling but instead offers a harmonious blend of both paradigms within the Bayesian framework.

In social sciences, where empirical analysis is often intertwined with complex theoretical constructs, the BPEN offers a robust alternative to traditional modeling techniques. It allows for the empirical validation of theoretical claims without succumbing to the perils of overfitting or underestimating the importance of theoretically significant predictors. As such, this approach aligns with the growing advocacy for methodological pluralism in the discipline, championing a modeling strategy that is both data-informed and theory-conscious.

While the Bayesian Partially-Protected Lasso (BPL) represents a significant methodological advancement, it is not without limitations. First, a key feature of the BPL is its reliance on theoretical guidance for selecting variables to protect, which ensures that the method aligns with substantive research objectives and preserves theoretically significant variables. However, this strength also introduces a potential challenge: if the protected variables are chosen based on poor theoretical justification or inconsistent criteria, the method's effectiveness may be compromised and may lead to suboptimal model performance. Second,the BPEN adds complexity to the modeling process–requiring custom prior specifications, tuning of multiple hyperparameters, and more computationally intensive sampling procedures, which may limit accessibility for practitioners unfamiliar with such techniques. Third, while the model offers credible intervals and posterior distributions, the partial protection mechanism introduces asymmetries in penalization that can complicate inference, especially when comparing protected and unprotected variables. Fourth, the trade-off between flexibility and model interpretability can present challenges, particularly in applications where the balance between empirical fit and theoretical adherence is difficult to maintain. For example, protecting variables might result in retaining predictors with weak explanatory power and might potentially complicate the narrative derived from the model. These considerations highlight the importance of careful theoretical reasoning to ensure the effective application of the BPL framework.Finally, in cases where theoretical guidance is absent or ambiguous, the distinction between protected and unprotected variables becomes less justifiable, potentially undermining the objectivity of the modeling process.

In the broader context of data-driven research, the BPEN represents a methodological innovation that does not merely refine the tools of analysis but embodies a commitment to integrating empirical rigor with theoretical depth. It challenges the conventional dichotomy between predictive and explanatory modeling, advocating for a synthesis that enhances both the accuracy of predictions and the relevance of explanations in social science research.

## Figures and Tables

**Figure A1.**
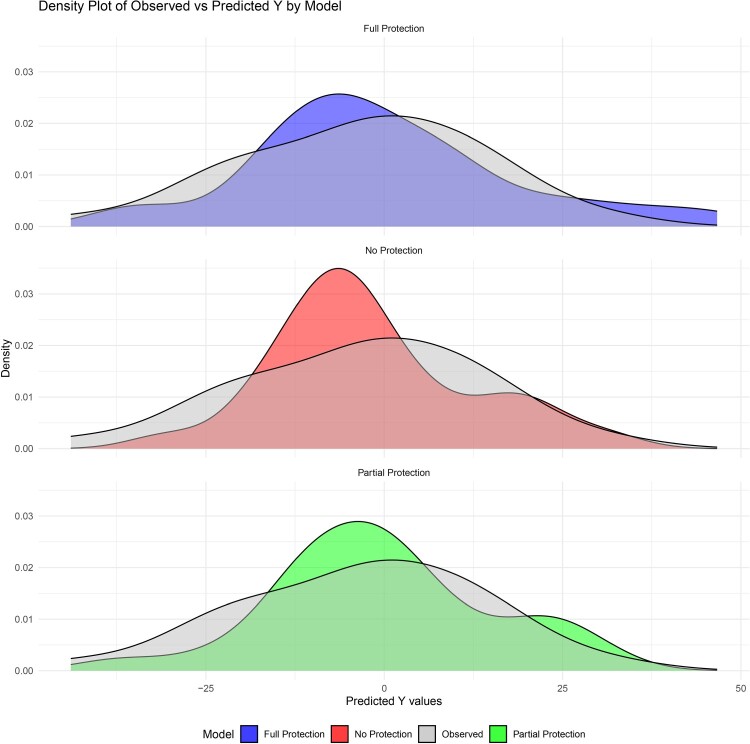
Posterior predictive checks: density plots of predicted versus actual outcomes for three variants of the Bayesian Lasso models.

## Data Availability

We used a simulated dataset as described in the article, and the panel survey data from ref. [6], which is publicly available. The code, and the data simulation process are available in our R package ProtectR at https://github.com/selimyaman/protectR.

## Appendix 1. Lasso Gibbs sampling steps

This appendix provides the specific MCMC steps required to estimate the Bayesian partially-protected Lasso as described in this research letter. Performing these steps iteratively many times produces empirical marginal posterior draws after convergence of the Markov chain can be asserted. All code is written in R and standard diagnostics, both graphical and formal, are used to assess the state of the chain with regard to its stationary distribution.

The Bayesian Lasso for interval measured outcomes with partially protected variables uses the standard semi-conjugate specification, and is estimated by the following sampling scheme:

**Update** β: $\beta |\tau^{2},\sigma^{2},Y∼N((\frac{1}{\sigma^{2}}X^{T}Y+\text{diag}(\frac{1}{\tau^{2}}){)}^{-1}X^{T}Y,\sigma^{2}(\frac{1}{\sigma^{2}}X^{T}X+\text{diag}(\frac{1}{\tau^{2}}){)}^{-1})$
**Update** $\tau^{2}$ For non-protected variables: $\tau_{\text{non-protected}}^{2}|\beta ,\sigma^{2},\lambda^{2}∼\mathrm{IG}(\sqrt{\frac{\lambda^{2}\sigma^{2}}{\beta_{\text{non-protected}}^{2}}},\lambda^{2})$
For protected variables: $\tau_{\text{protected}}^{2}|\beta ,\sigma^{2}∼\Gamma (\alpha +\frac{1}{2},\beta +\frac{\beta_{\text{protected}}^{2}}{2\sigma^{2}})$
**Update** $\sigma^{2}$: $\sigma^{2}|\beta ,\tau^{2},Y∼\mathrm{IG}(\frac{n-1+p}{2},\frac{1}{2}(Y-X\beta {)}^{T}(Y-X\beta )+\frac{1}{2}\beta^{T}\text{diag}(\frac{1}{\tau^{2}})\beta )$
**Update** $\lambda^{2}$: $\lambda^{2}|\tau_{\text{non-protected}}^{2}∼\Gamma ((p-n_{\text{prot}})+a,\frac{1}{2}\sum_{\text{non-protected}}\tau_{j}^{2}+b)$

The Bayesian Lasso for dichotomous outcomes with partially protected variables uses the following Gibbs sampling sub-steps:

**Update** $\omega^{2}$: $\omega^{\left(t\right)}∼p(\omega \;|\;\beta^{\left(t-1\right)},\sigma ,\nu )$
**Update** *λ*: $\lambda^{\left(t\right)}∼p(\lambda \;|\;X,\beta^{\left(t-1\right)},\kappa )$
**Update** *z*: $z^{\left(t\right)}∼p(z\;|\;X,\beta^{\left(t-1\right)},\lambda^{\left(t\right)},\kappa )$
**Update** β: $\beta^{\left(t\right)}∼p(\beta \;|\;X,z^{\left(t\right)},\lambda^{\left(t\right)},\omega^{\left(t\right)},\nu ,\sigma ,\kappa )$
**Update** *ν*: $\nu^{\left(t\right)}∼p(\nu \;|\;\beta^{\left(t\right)},\sigma ,\kappa )$
**Calculate** the log posterior probability: ${\text{lpost}}^{\left(t\right)}=\log p(y,X,\beta^{\left(t\right)},\nu ,\kappa ,\sigma )$

Then after the last step the parameter values above at the iteration with the highest posterior probability are returned to complete a single iteration of the Gibbs sampling process. For an interesting and important comparison of Gibbs sampling strategies in this context see [3]. This work also demonstrates the importance of Lasso estimation from a Bayesian approach.

## Appendix 2. Elastic net MCMC procedure

We employed a Markov chain Monte Carlo (MCMC) procedure to estimate three Bayesian linear regression models in simulated dataset: (1) a *fully protected* model, which applies no regularization to any predictor; (2) a *partially protected* model, which shrinks all but a predefined subset of predictors; and (3) an *unprotected* model, in which all predictors are regularized. To select the optimal strength of the elastic net penalty, we performed *k*-fold cross-validation (CV) with *k* = 5 over a grid of $\lambda_{1}$ (L1) and $\lambda_{2}$ (L2) values, recording the mean squared error (MSE) of predictions on the held-out folds. We then refit each model on the entire sample using the best $\left(\lambda_{1},\lambda_{2}\right)$ pair identified by CV. The pairs identified during the CV can be found in the Appendix Section 3.

Formally, the observations satisfy

$$Y_{i}=\alpha +\sum_{k=1}^{K}\beta_{k}\;X_{\mathrm{ik}}+\varepsilon_{i},\;\varepsilon_{i}\;∼\;N(0,\sigma^{2}),$$

for i=1,…,N, where $X_{\mathrm{ik}}$ denotes the value of the *k*th predictor for the *i*th observation, and $\alpha ,\beta_{k},\sigma$ are unknown parameters. To encourage sparsity in coefficients for which shrink[k]=1, we place a Laplace (double-exponential) prior on $\beta_{k}$ with scale parameter $\lambda_{1}$, along with a Gaussian penalty that contributes a term $-\frac{1}{2}\;\lambda_{2}\;\beta_{k}^{2}$ to the log-posterior. If shrink[k]=0 (i.e. the predictor is ‘protected’), we instead assign a weakly informative prior $\beta_{k}∼N(0,10,000)$. Summarizing these choices, the negative log-posterior contribution for each $\beta_{k}$ subject to shrinkage takes the form

$$-\log p(\beta_{k})=\frac{|\beta_{k}|}{\lambda_{1}}+\frac{\lambda_{2}}{2}\;\beta_{k}^{2},$$

while unshrunk coefficients only incur the weakly informative Gaussian prior term.

We used the No-U-Turn Sampler (NUTS) implemented in Stan to draw posterior samples, relying on 4 chains of 500 total iterations each (half of which serve as warm-up). We set seed =123 for reproducibility. At each iteration (and in each fold of the cross-validation), the sampler updates $\left(\alpha ,\beta_{1},\ldots ,\beta_{K},\sigma \right)$ according to the joint posterior,

$$\begin{array}{rl} & p(\alpha ,\beta ,\sigma |Y)\propto \prod_{i=1}^{N}N\;\left(Y_{i}|\alpha +x_{i}^{⊤}\beta ,\;\sigma^{2}\right)\\ & \;\times \prod_{k:\;\mathrm{shrink}[k]=1}\left[\mathrm{Laplace}(0,\lambda_{1})\times \mathrm{Gauss}\left(0,\lambda_{2}^{-1}\right)\right]\times \prod_{k:\;\mathrm{shrink}[k]=0}\mathrm{Gauss}\left(0,10,000\right). \end{array}$$

where $x_{i}$ denotes the row of predictors for observation *i*. We then use the posterior means or medians of β and *α* from each fold to predict the held-out data and compute an MSE. After identifying the $\left(\lambda_{1},\lambda_{2}\right)$ combination that minimizes out-of-sample MSE, we refit the model on the full data set to obtain final posterior draws. We repeat this process for the fully protected, partially protected, and unprotected models, thereby yielding three distinct sets of posterior estimates for comparison.

## Appendix 3. Posterior prediction

We evaluate the performance of our Bayesian Partially Protected Lasso models in the simulation using posterior predictive checks, in addition to comparing the models in Table A1 based on the posterior estimations' error rates. This evaluation is crucial for assessing the models' accuracy in reproducing observed data characteristics within new datasets. By extracting posterior samples and simulating outcomes based on these distributions, we generate robust predictive data points for each model variant–Full Protection, Partial Protection, and No Protection. We replicate the outcome predictions 1000 times, using the posterior mean of the model coefficients and incorporating normally distributed noise that reflects the estimated residual variance.

**Table A1.** Best combination of L1 ( $\lambda_{1}$) and L2 ( $\lambda_{2}$) for each model and dataset.

Model	Dataset	$\lambda_{1}$ (L1 – Lasso)	$\lambda_{2}$ (L2 – Ridge)
Fully Protected	Simulation	1.05	2.000
Partially Protected	Simulation	0.10	0.575
No Protection	Simulation	0.10	0.575
Fully Protected	Liberia Reanalysis	2.00	0.575
Partially Protected	Liberia Reanalysis	0.10	0.575
No Protection	Liberia Reanalysis	0.10	0.575

To assess the model fit and predictive accuracy visually, we create kernel density plots in Figure A1, comparing the predicted outcomes to the actual observed outcomes from the test set. It is evident that all three models generate similar values for the unseen dataset, although the No Protection model performs slightly worse compared to the Partially Protected and Fully Protected models.

## Appendix 4. Regularization parameters

Here we summarize the final penalty hyperparameters ( $\lambda_{1}$ and $\lambda_{2}$) chosen for each model across both the simulation dataset and the Liberia reanalysis. These values were determined via *k*-fold cross-validation, searching over a grid of candidate $\lambda_{1}$ and $\lambda_{2}$ combinations, then selecting the pair that yielded the lowest mean squared error (MSE).

*Parameter Selection Procedure*. We conducted a grid search over candidate values of $\lambda_{1}$ and $\lambda_{2}$ to minimize the mean squared error (MSE) via *k*-fold cross-validation (*k* = 5). Specifically, $\lambda_{1}$ corresponds to the L1 (lasso) regularization penalty, and $\lambda_{2}$ corresponds to the L2 (ridge) penalty. Both parameters were searched over the following grid:

$$\lambda_{1},\lambda_{2}\in \{0.1,0.575,1.05,1.525,2.0\}.$$

The best combination for each model (fully protected, partially protected, and no protection) was identified based on the lowest cross-validated MSE. These values are summarized in Table A1.
